# Investigation of Field Emission Properties of Carbon Nanotube Arrays of Different Morphologies

**DOI:** 10.3390/nano14090763

**Published:** 2024-04-26

**Authors:** Maksim A. Chumak, Alexander V. Shchegolkov, Eugeni O. Popov, Sergey V. Filippov, Anatoly G. Kolosko, Aleksei V. Shchegolkov, Arif A. Babaev

**Affiliations:** 1 Centre of Nanoheterostructure Physics, Ioffe Institute, Saint Petersburg 194021, Russia; 2 Institute of Power Engineering, Instrumentation and Radioelectronics, Tambov State Technical University, Tambov 392000, Russia; 3 Division of Plasma Physics, Atomic Physics and Astrophysics, Ioffe Institute, Saint Petersburg 194021, Russia; e.popov@mail.ioffe.ru (E.O.P.); s.filippov@mail.ioffe.ru (S.V.F.); agkolosko@mail.ru (A.G.K.); 4 Center for Project Activities, Moscow Polytechnic University, Moscow 107023, Russia; alexxx5000@mail.ru; 5 Institute of Physics DFIC RAS, Makhachkala 367015, Russia; babaev-arif@mail.ru

**Keywords:** atomic layer deposition, nanoisland films, direct-current plasma-enhanced chemical vapor deposition, thin-layer structures, carbon nanotubes, field emission

## Abstract

This article presents, for the first time, a comparative analysis of the emission characteristics of large-area field-effect cathodes (LAFE) based on carbon nanotubes (CNTs) of various morphologies according to key parameters using a unique computerized technique. The work presents a description of a technology for creating various CNT arrays and their comprehensive structure characterization. All CNT arrays synthesized by the catalytic PECVD method on a silicon substrate showed a high degree of chemical purity under the presented technological conditions. In some cases, nanoisland films of Fe were used as a catalyst; in others, thin films of NiO were used, which were deposited on a silicon wafer by chemical vapor deposition (CVD) and atomic layer deposition (ALD), respectively. As a result of these studies, it turned out that an array with a thick CNT coating has good resistance to the action of strong electric fields, fairly good uniformity of distribution of emission centers, a fairly high selection current (2.88 mA/cm^2^ at 4.53 V/μm), and compliance with the normal current mode according to the “orthodox” test, which makes the morphology of such structures the most promising for further technological optimization of CNT-based cathodes for various practical applications.

## 1. Introduction

Carbon nanotubes (CNTs) have emerged as highly promising materials for the production of micro-sized devices. One particular application that stands out is the development of field emission-based electron sources. These sources have the potential to be utilized in a wide range of devices, such as photoelectric converters, electron nanolithography systems, amplifiers of electrical signals (traveling-wave tubes), monitors, X-ray machines, lamps, gas sensors, space telescopes, and microscopes [1,2]. They are also employed in scanning electron microscopy (SEM) and transmission electron microscopy (TEM) [3]. Large-area field emitters (LAFE) that are based on CNTs demonstrate a low threshold field [4], making them highly efficient in generating high-emission currents. For instance, a single-walled carbon nanotube (SWCNT) can produce emission currents of approximately 2 µA [5]. A low threshold voltage for field emission, approximately 1–3 V/µm, has been observed in SWCNT films [6]. This can be attributed to the unique properties of CNTs, including their large aspect ratio, high conductivity, and thermal stability [7]. As a result, CNTs have been utilized in a variety of field emission applications, such as lighting elements [8], disinfecting ultraviolet lamps [9], and touch and pressure sensors [10]. Furthermore, several research groups have successfully developed field emission cathodes based on CNTs for the miniaturization of ion sources. These include electron ionization sources and chemical ionization sources used in portable mass spectrometers [11,12]. The high electrical conductivity and excellent electron emission characteristics of CNTs make them ideal candidates for such applications. One notable application of field emission cathodes is in commercial tomographs that utilize carbon nanotubes. These tomographs feature a linear array of X-ray sources based on CNTs, which enables the collection of projection images without the need to move the X-ray source [13]. For an effective field emitter, it is crucial to have a high and stable current density [14]. The field emission current is influenced by the types of CNTs and the methods used for their synthesis. The crystallinity of CNTs plays a significant role in determining factors such as the Joule heating, stability, emission current density [15], and uniformity of current transfer [16]. Additionally, the emission characteristics of field cathodes are strongly influenced by the morphology of CNT arrays and the geometric parameters of individual nanotubes [17,18]. These factors need to be carefully considered and optimized to achieve optimal performance in field emission devices.

Today, there are many methods to grow CNTs on various substrates [19]. Among them are the electric arc methods [20,21], laser ablation [22], and chemical vapor deposition (CVD) [23,24]. It is known that the DC PECVD (direct-current plasma-enhanced CVD) method is the most reliable method with the best controllability of the geometrical parameters for growing CNT arrays [25]. This method gives the best indicators of the verticality and correctness of the CNTs’ shapes, regardless of the density of their nucleation, as well as the perfection of the structure of the CNTs’ walls and the level of their contamination with foreign phases. For example, in [26], it is shown that, using the thickness of the catalyst film deposited on a flat substrate, it is possible to control a CNT’s diameter.

One of the most ordinary and morphologically simple CNT-based emitters is a thick array of non-oriented “spaghetti-shaped” nanotubes. In [27], the authors show that to achieve a current density of 1 μA/cm^2^, a field of 2.6 V/μm is required for an array grown at 450 °C, and one of 3.7 V/μm is required for an array grown at 500 °C. To ensure a current density of 1 mA/cm^2^ for CNTs grown at 450 °C and 500 °C, fields of 3.5 V/μm and 5.54 V/μm, respectively, are required. The peak value of the current density was 7 mA/cm^2^ at an electric field strength of 5.24 V/µm for the cathode current indicated in [28]. Another paper [29] shows a comparison of arrays of this type obtained under different technological growth conditions. The most successful variants of the structures had a turn-on field of 2.5 V/µm and an effective field enhancement factor *β* of 6200–7800. The influence of different growth conditions on the emission characteristics of the same arrays of CNTs was shown in [30]. The best result showed that the threshold and turn-on electric fields were 2.48 and 3.98 V/µm, respectively, and the emission current density reached 30 mA/cm^2^. A CNT layer 185 µm thick in [31] showed an *E_on_* value of 0.67 V/µm, and the applied field to extract a 1 mA current was 1.52 V/µm and *β* = 3976.

Another array of CNTs frequently encountered in the literature is long densely spaced vertically aligned carbon nanotubes (VACNTs). A case with a very high density of tubes showed that there were practically no emission centers over the entire area of the cathode; instead, the centers were located at the edges of the array, which is direct experimental evidence of the “edge effect” [32]. This type of array in the [33] showed that the turn-on electric field and filed required for obtaining an emission current density of 10 mA/cm^2^ were 2.3 and 5.1 V/µm, respectively. In another article [34], the VACNT cathode showed a turn-on field of 0.66 V/µm and an emission current of about 1.5 mA (85 mA/cm^2^) at an applied field of 1.5 V/µm. The nanotubes were grown using hot-filament CVD.

Among the literature, quite often, there are cathode structures with short, densely arranged CNTs. In ref. [35] CNTs were grown on stainless-steel substrates. The field required to obtain an emission current density of 1 mA/cm^2^ was 8.3 V/m. The field enhancement factor was estimated to be 1140. The work of Neupane et al. [36] showed that, with a decrease in the CNT arrangement density, it is possible to reduce the fields necessary for the emission to occur. The electric field corresponding to an emission current density of 1 µA/cm^2^ for high-density CNTs was 7.96 V/µm. The field decreased to 5.19 as the density of the mutual arrangement of the tubes decreased. Similar results were obtained in [37].

Excellent emission performance is possessed by emitters with vertically aligned CNTs located discretely relative to each other. In such systems, it was found that an increased density of emission sites and the best degree of current uniformity over the area were achievable [38]. The CNT density was controlled by a third electrode in the form of mesh in a triode PECVD. The dense CNTs showed a turn-on field of 3 V/µm and a current density of 0.2 mA/cm^2^ at 3.5 V/µm. The CNTs grown under optimal mesh bias had the lowest turn-on field and the highest current density of 0.6 V/µm and 1.5 mA/cm^2^, respectively, at 2.7 V/µm.

The literature compares the emission characteristics of CNT arrays obtained under different conditions, with different cathode areas and interelectrode spacing, which makes it difficult to compare them with each other [27,28,29,30,31,32,33,34,35,36,37,38]. Therefore, for a clear comparison of the field emission characteristics of various morphologies of CNT arrays according to a series of criteria, this work presents, for the first time, simple and common morphologies of CNT arrays grown on a silicon substrate by the DC PECVD method in the presence of catalysts. Among the criteria for the effectiveness of field emission, certain threshold field emission voltages, emission currents, numbers and levels of uniformity of the distribution of emission centers over the cathode area, and levels of compliance with the normal current regime according to the “orthodox” field emission test were accepted. Field emission studies were carried out using a multichannel data recording system, which made it possible to identify the most efficient configurations of CNT arrays potentially suitable for wide use.

## 2. Materials and Methods

### 2.1. Growing of CNTs

**Figure 1:**
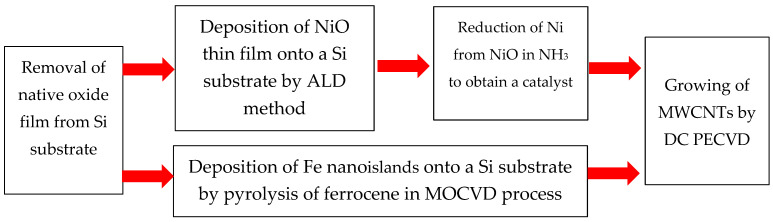
Diagram of CNT production.

Preparation of Si substrates: Before growing CNT structures, a KEF 7.5 grade Si substrate was subjected to a cleaning process. Firstly, it was cleaned of natural oxide by treating it with HF acid. Then, it was thoroughly washed with distilled water to remove any residues of the etching products. Finally, it was boiled in an acetone solution to eliminate any organic contaminants. To fabricate the structures presented in this work, we used two different approaches. One of them was the growth of nanotubes on an Fe catalyst, and the other is the growth of CNTs on the a NiO film. All the substrates had squares of 1 cm^2^. A generalized diagram explaining different growth patterns of CNT arrays is presented in Figure 1.

**Figure 2:**
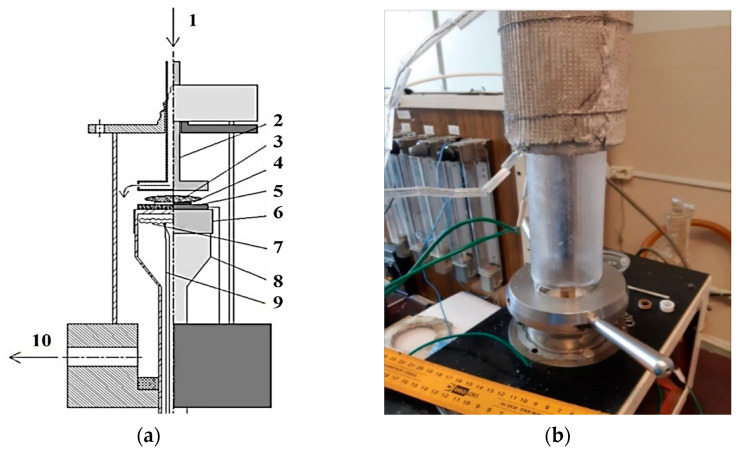
(**a**) Diagram of the DC-PECVD setup for growing of CNTs: 1—gas mixture supply, 2—anode, 3—plasma, 4—substrate, 5—cathode, 6—tantalum shield, 7—heater, 8—pedestal, 9—thermocouple, 10—vacuum pump. (**b**) Photograph of the DC-PECVD setup for growing CNTs on flat substrates with a gas distribution system.

Deposition of Fe nanoislands. In the case of growing CNTs on an Fe catalyst, we used ferrocene (bis (η-cyclopentadienyl) iron) (Sigma-Aldrich, St. Louis, MO, USA, 98%), which serves as a source of metal in the metalorganic chemical vapor deposition (MOCVD) process for producing Fe nanoislands on a Si substrate. The reactor for the deposition of Fe nanoparticles through the pyrolysis of ferrocene is shown in Figure 2, in which anode 2 was removed to implement the MOCVD process. The evolved reagent vapors are transferred by argon flow to the underlying deposition area, where a quartz pedestal with a built-in heater is located. There is a graphite washer 45 mm in diameter with the substrates, which were polished KEF-5 (100) silicon wafers without a natural SiO_2_ layer. For the sample presented in this work, deposition was carried out for 30 min at a pedestal temperature of 700 °C and a total pressure of 700 Pa. The consumption values of Ar (99.999%) and ferrocene were 50 and 0.27 sccm, respectively. No morphological features were observed on the smooth surface of the obtained Fe layer (with a nominal thickness of ≈30 µg/m^2^). This approach was used to obtain the structures C1 and C2.

Deposition of NiO film. During the growth of CNTs on the Ni catalyst, the NiO precipitation process took place in a Picosun R-150 atomic layer deposition (ALD) reactor with a closed configuration featuring hot walls. NiCp_2_ (Sigma-Aldrich, 99.99%) and O_3_-H_2_O were selected as precursors for the growth of NiO, while high-purity N_2_ (99.999%) served as both the carrier and purge gas. NiO was deposited through the sequential exposure of CNTs to NiCp_2_ and O_3_-H_2_O, with pulse durations of 1 s and 6 s, respectively, and a 10 s N_2_ gas purge time. A single cycle consisted of NiCp_2_/purge/O_3_/purge = 1.0/10.0/6.0/10.0 s. The deposition temperature remained at 250 °C, with a sublimation temperature of NiCp_2_ at 110 °C. A similar growth approach was employed in previous studies [39,40]. The initial NiO film thickness for enhancing CNT growth ranged from 3.5 to 3.9 nm [41]. The formal increase per cycle was about 0.4 Å. The thickness of the deposited NiO film was 3.8 nm, which corresponds to a number of ALD cycles equal to 95. This approach was used to obtain the structures C3 and C4.

Reduction of Ni from NiO. Immediately before growing CNTs in the DC PECVD reactor, the NiO coating was subjected to heat treatment in an ammonia-based atmosphere through sequential heating to a temperature of 680 °C and holding for 5 min. This led to the formation of a layer of individual nickel metal nanoparticles. A reducing atmosphere was created in a working mixture of NH_3_ (10 sccm) and Ar (sccm) at a total pressure of 300 Pa by the catalytic decomposition of ammonia on the surface of a layer of NiO and reduced Ni.

Synthesis of CNTs. Throughout the CNT arrays’ growth via the DC PECVD technique, the reactor featured a system of electrodes (illustrated in Figure 2): a graphite washer functioned as the cathode, while a stainless-steel disk 2 (ø 45 mm) served as the anode. The distance between these electrodes measured 40 mm. The samples discussed herein were produced through a deposition process lasting 10 min, conducted at a pedestal temperature of 740 °C and a total pressure of 300 Pa. The operational environment comprised ammonia, supplied at a flow rate of 200 sccm, alongside acetylene at 100 sccm. Samples obtained after Fe or NiO deposition served as substrates. The discharge was characterized by a current of 7.5 mA and an anode voltage of 480 V. A similar growth method was used in [42,43,44].

### 2.2. Research Methods

Scanning electron microscopy (SUPRA 55-25-78 microscope) was used to analyze the results of the growth of the CNT arrays. Transmission electron microscope (TEM) microphotographs were obtained by a Carl Zeiss Libra 200FE device. Raman spectroscopy was performed to identify CNTs and determine the degree of their defectiveness and crystalline quality on a Renishaw Raman spectrometer using 580 nm green laser excitation with a pinhole of 1 mm and exposure time of 100 s. The XPS measurements were conducted at the “Physical Methods of Surface Investigation” Resource Center of St. Petersburg State University using an “Escalab 250Xi” photoelectron spectrometer with AlKα radiation (photon energy 1486.6 eV). Spectra were recorded in the constant pass energy mode at 100 eV for the survey spectrum and 50 eV for the element core level spectrum, using an XPS spot size of 650 μm. The total energy resolution of the experiment was about 0.3 eV. Investigations were carried out at room temperature in an ultrahigh vacuum in the order of 1 × 10^−9^ mbar. An ion–electronic charge compensation system was used to neutralize the sample charge.

The study on field emission was carried out using a computerized method that involved multichannel data collection and online processing of the field emission data [45]. In this method, flat electrodes were utilized, along with a fast high-voltage scanning regime. The distance between the electrodes was 370 μm, and the measuring chamber was maintained under technical vacuum conditions (10^−7^ Torr).

## 3. Results and Discussion

### 3.1. Sample Characterization

**Figure 3:**
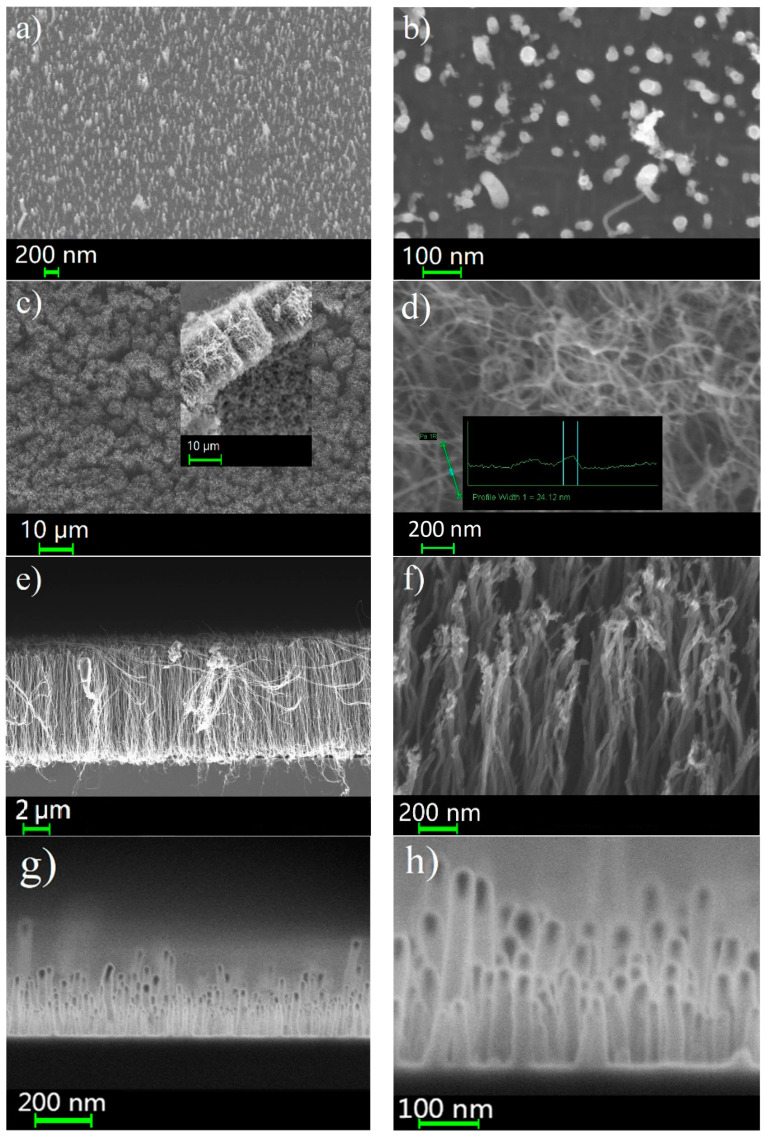
SEM images of different carbon nanotube arrays: C1—discrete vertically aligned short CNTs (**a**) (detailed image (**b**)); C2—thick cover (**c**) (detailed image (**d**)); C3—dense array with vertically aligned long CNTs (**e**) (detailed image (**f**)); C4—dense array with short CNTs (**g**) (detailed image (**h**)).

Figure 3 shows SEM images of a general view of the different cathodes used in this study. Sample C1 is CNTs discretely spaced from each other with a placement density of ~1.7 × 10^10^ cm^2^ (Figure 3a). The vast majority of CNTs are oriented vertically, and have a slightly conical shape and a small spread in height (Figure 3b). Their average length is more than 200 nm. Fe catalyst particles are observed at the free ends of the tubes. Sample C2 is a thick coating of CNTs (Figure 3c). The inset shows a cross-section of the array, from which the thickness is estimated to be 20 µm. The tubes have an inhomogeneous diameter distribution with an average value of 12 nm (Figure 3d), and Fe catalyst particles are seen at the ends. Sample C3 is a continuous dense CNT array (Figure 3e). The height of its tubes is 7 µm. Figure 3f shows a detailed image of them, which shows a uniform distribution of tubes over a diameter of 10 nm. Figure 3g shows an SEM image of a general view of sample C4. In comprises a dense array of short CNTs with an average length of 300 nm. A more detailed image of the structures is shown in Figure 3h. It should also be noted that there are rather high tubes in the array, which are at least twice as long as the rest of the mass of tubes. According to the SEM images, all CNTs have a Ni catalyst in their heads.

**Figure 4:**
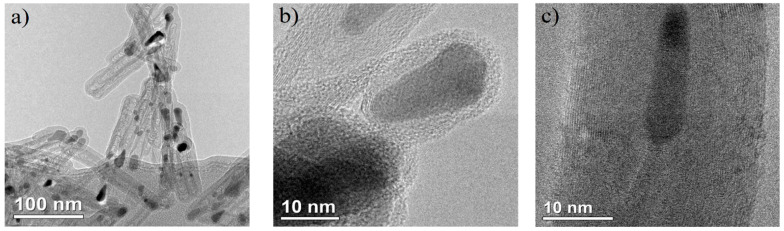
TEM images of (**a**) general view of CNTs placed on a carbon grid, (**b**) Ni catalyst particles in the tops of nanotubes, and (**c**) internal structure of CNTs.

Transmission electron microscopy (TEM) analysis of the carbon nanotube (CNT) arrays synthesized via our approach revealed the consistent presence of CNTs exhibiting uniform structures across all samples. As depicted in Figure 4a, the internal composition exhibits discernible imperfections, attributed to catalyst particle traversal during growth, manifesting as defects in the inner walls. Further scrutiny of Figure 4b unveils elongated Ni catalysts situated at the tube termini, exhibiting a singular crystalline configuration, evident from the discernible periodicity in Ni atom alignment. Additionally, Figure 4c quantifies the number of nanotube layers, which peaks at 30.

**Figure 5:**
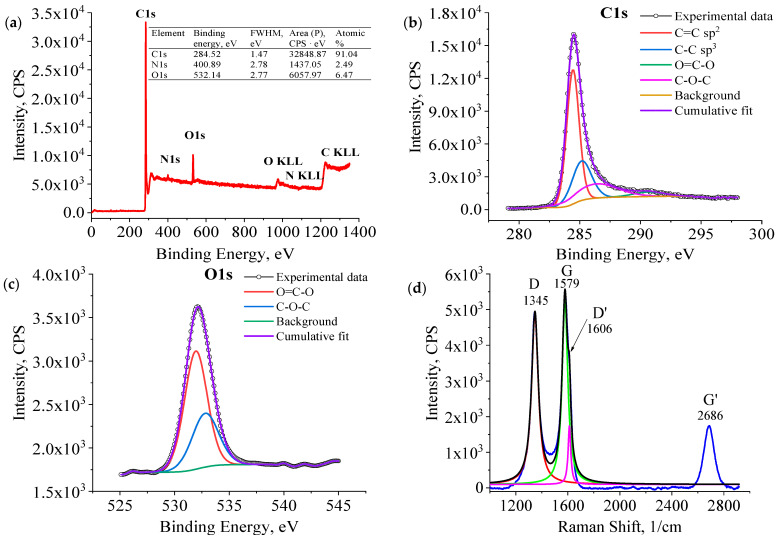
XPS Survey (**a**); HRXPS of C1 (**b**) and O1 (**c**) peaks for sample C3; (**d**) Raman shift of CNTs grown on Si substrate (C2), which was decomposed into components: D (red line), G (green line) and D’ (magenta line).

To gain a comprehensive grasp of the structural composition of carbon nanotubes (CNTs) generated through the existing PECVD method, X-ray photoelectron spectroscopy (XPS) analyses were conducted to scrutinize the elemental chemical states within the synthesized materials. Employing the XPS technique for the selective sample examination of CNTs (C3), it was revealed that carbon accounted for 91.04% of the composition, with only 6.47% oxygen and 2.49% nitrogen, indicating a notably high level of purity in the resultant nanotubes under the operational parameters of the current fabrication process (refer to Figure 5a). Further examination of the C1 peak (as depicted in Figure 5b) unveiled distinct bonds, including sp^2^ C=C (284.44 eV), sp^3^ C-C (285.16 eV), C–O–C (286.35 eV), and O=C-O (290.68 eV). Notably, the analysis indicated the absence of a Pi-plasmon loss peak within the CNT structure. Additionally, the O1 peak (as illustrated in Figure 5c) exhibited two components with binding energies corresponding to C–O–C (532.80 eV) and O=C-O (531.94 eV), aligning with the outcomes derived from the deconvolution of the C1 peak.

Raman spectroscopy is a valuable tool for analyzing carbon-based materials [46] and was utilized to identify CNTs in this study. The D band at 1345 1/cm is linked to structural defects, while the G band at 1579 1/cm corresponds to the in-plane stretching vibration of carbon–carbon bonds within graphene sheets (Figure 5d). A faint shoulder at a higher wavelength on the G peak (D’, 1606 1/cm) indicates structural abnormalities. Raman spectroscopy provides insights into the level of defects and crystal quality of the material [47,48]. The intensity ratios of certain bands in the spectrum reveal details about defect quantity and carbon atom content in the CNT samples. This method also allows for the assessment of the graphitic order and crystal quality of nanotubes. The ratios of the D/G, G’/G, and G’/D bands are 0.82, 0.34, and 0.29, respectively. The emphasis is on the importance of the ID/IG peak ratio in CNT structure analysis. A high ratio suggests numerous defects, while lower values of IG’/ID and IG’/IG ratios indicate a relatively well-structured crystal and limited graphitic order distribution.

### 3.2. Field Emission Study

**Figure 6:**
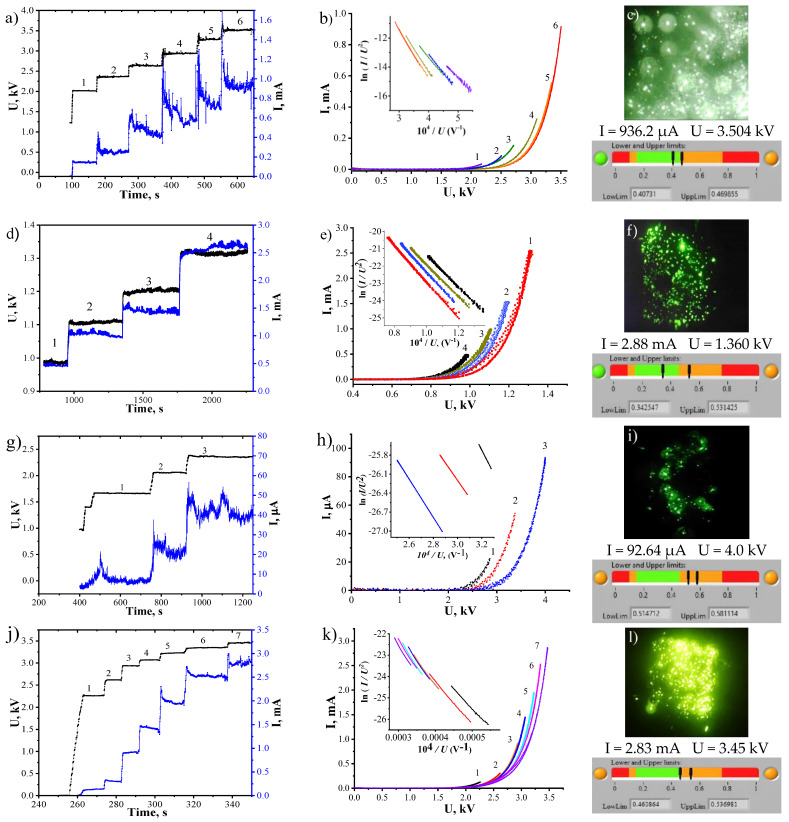
Training of the cathodes by voltage steps for C1 (**a**), C2 (**d**), C3 (**g**), and C4 (**j**); IVCs for C1 (**b**), C2 (**e**), C3 (**h**), and C4 (**k**) (each IVC number corresponds to the step number) and corresponding IVCs in Fowler–Nordheim coordinates (in inserts); glow patterns from a phosphor screen for C1 (**c**), C2 (**f**), C3 (**i**) and C4 (**l**) and the results of the “orthodox” tests (in inserts).

During the initial phase of field emission measurements, the sample underwent a high-voltage training process. However, some of the most unstable sites that were protruding significantly above the surface were also eliminated. The time-dependent behaviors of the voltage pulses and emission current pulses (voltage and current levels) are depicted in Figure 6a,d,g,j.

The emission currents recorded during training peaked at 916.6 µA and 92.6 µA for C1 and C3, respectively, while C2 and C4 reached 2.88 mA and 2.83 mA, correspondingly. Figure 6 illustrates the stepwise progression of current over time, demonstrating a saturation phenomenon where the current stabilizes at a constant level after each voltage increment. According to the findings from [5], this saturation suggests an adsorption effect, typically absent in pristine nanotubes. The observed saturation in the emission current arises from a combination of field- and current-induced reductions in the tunneling amplification of adsorbate states. The emission current characteristics are depicted in Figure 6b,e,h,k with respect to the applied voltage (IVC), acquired using rapid voltage sweeping at every increment of the step-wise current dependency (one IVC per 20 milliseconds). IVCs were gauged when the current reached saturation, where each voltage step aligns with a specific IVC. The current at each IVC was logged until the voltage level corresponding to the respective step was reached. Additionally, all IVCs are illustrated as Fowler–Nordheim (FN) coordinates within the insets. The IVCs were approximated by the FN equation in the Elinson–Schrednik annotation [49]:

(1) $$I=A_{eff}\frac{\alpha_{FN}}{1.1}\varphi^{-1}\alpha_{eff}^{2}U^{2}exp\left(1.03\eta \right)exp\left(-0.95b_{FN}\varphi^{\frac{3}{2}}\frac{1}{\alpha_{eff}U}\right)$$

(2) $$\beta_{eff}=\alpha_{eff}d_{sep}$$

where $\eta ={b_{FN}\varphi }^{3/2}/F_{R}=b_{FN}{c_{S}^{2}\varphi }^{-1/2}$, *F_R_* = *φ*^2^*c_S_*^−2^ represents the barrier removal field, *c_S_*^2^ = 1.439965 × 10^−9^ eV^2^m/V is the Schottky constant, *a_FN_* = 1.541433 × 10^−6^ [A∙eV/V^2^] and *b_FN_* = 6.830890 × 10^9^ [eV^−3/2^∙V/m] are the first and second Fowler–Nordheim constants, *φ* is the emitter work function [eV], *β_eff_* is the effective field enhancement factor at the emitter tip, *A_eff_* is the effective emission area, U is the applied voltage, and *d_sep_* is the interelectrode distance. The work function for CNTs was assumed to 4.6 eV [50].

As a result of the IVC approximation procedure, the effective field enhancement factors *β_eff_* of the emitters were determined. The turn-on field, *E_on_*, is defined as the applied electric field required to obtain an emission current density of 10 µA/cm^2^ from the emitters, and for the threshold field *E_th_*, it is 1 µA/cm^2^. Below are the parameters for IVCs with the highest recorded current output under the numbers 6, 4, 3, and 7 for samples C1, C2, C3, and C4, respectively. Sample C1, representing ultrashort tubes, showed a turn-on field *E_on_* of 8.1 V/µm, a threshold field *E_th_* of 6.5 V/µm, a maximum recorded current density *J_max_* of 916.6 µA/cm^2^, and a field enhancement factor *β_eff_* of 1100. For discrete CNTs, a current density of 1.5 mA/cm^2^ was also observed in [38], which is very close to our result. Sample C2 had the lowest turn-on field (*E_on_* = 3.0 V/µm) and threshold field (*E_th_* = 2.3 V/µm), the maximum recorded current density (*J_max_* = 2.88 mA/cm^2^), and the largest effective field enhancement (*β_eff_* = 2821) of all the structures. The sample was tested only up to 1200 V, since the experiment had to be interrupted due to the fact that “edge effects” began to appear on the structure. The total current in this case would no longer be correct when referring to the CNT current. It is quite obvious that with such characteristics, it is possible to obtain a current density from 1 to 10 mA/cm^2^ at high pulling fields on this type of CNT array. The structures shown in [27] also have low threshold and turn-on fields. For sample C3, the emission’s currents and its other characteristics turned out to be the worst of the structures considered. Its turn-on field *E_on_* = 9.8 V/µm and threshold field of *E_th_* = 9.0 V/µm turned out to be the largest among the presented arrays, and the maximum recorded current density was only 93.5 µA/cm^2^, with a relatively low *β_eff_* of 617. However, the tube arrays of the same type shown in the papers [33,34] have lower threshold and turn-on fields and higher emission currents. It is possible that this difference is due to distinctions in the density of the mutual arrangement of the CNTs, the radii of their curvature, or even the state of the tops. The switch-on field for sample C4 was *E_on_* = 6.1 V/µm, the threshold field *E_th_* = 4.8 V/µm, the maximum recorded current density *J_max_* = 2862.9 mA/cm^2^, and the *β_eff_* turned out to be 1380, which agrees at least with the geometric characteristics of the tips. Structures of a similar type in [35,36,37] required smaller fields to obtain a current density of 1 mA/cm^2^, when a field of 10.53 V/µm must be applied to sample C4. For clarity, the emission parameters are listed in Table 1.

Figure 6c,f,i,l shows the glow patterns of the phosphor screen for each sample at a maximum registered current level. The patterns show the number and arrangement of the emission centers over samples. Software processing of the glow patterns revealed approximately 600 sites for sample C1, 1070 for C2, and approximately 1210 for C4 when the average number of emission centers on sample C3 was only 60. Such a difference in the number of centers explains why samples C1 and C4 recorded higher levels of emission currents compared to C3. Obviously, the number of centers depends on the morphological features of the structures. In denser arrays of tubes, there are fewer emission centers, since the mutual screening effect reduces the level of the electric field at the tops of the tips, and therefore, the number of tips capable of emitting current decreases, while on more diluted arrays, the fields focused on CNTs are higher, and, thus, a larger number of tubes have the ability to lower their potential barrier to enable the release of electrons. Perhaps this explains why only a small percentage of CNTs are involved in the emission process. As for the C3 sample, it is likely that even with a small number of activated emission centers during high-voltage training, it is able to provide more current at increased voltages, and an increase in their number with a further increase in the field is not excluded. Samples C1, C2, and C4 show a fairly uniform distribution of centers over their entire area. Similar homogeneity was shown for a similar type of cathode in [38], but C3 is very heterogeneous in current output, due to the small number of operating centers. The work in [27] shows similar inhomogeneity in the distribution of emission centers with cathode morphology as in sample C2. At the same time, the brightness of the glow on all samples varies greatly due to the strong difference in the height of the nanotubes; the highest ones give more current, since the focus of the electric field is stronger on them. To achieve uniformity in current output, it is necessary to manufacture structures with a minimum spread in the height of the tips.

Next, we checked whether the current mode of the samples corresponded to the classical law of field emission using the Forbes test (so-called “orthodox” test), a detailed description of which is contained in [51]. To pass the test, it is necessary that the value of the dimensionless electric field *f* does not exceed the upper limit of the permissible range with the corresponding work function for the CNTs. Samples C1 and C2 successfully passed the test, so the electron emission in the experiments corresponds to the FN law, as the dimensionless field *f* = 0.4 does not exceed the value *f* set for CNTs (for the work function 4.6 eV) equal to 0.45 (insert in Figure 6c,f). This means that the specified electric fields for these structures lie within acceptable ranges and correspond to normal current operation. However, as can be seen from the processing of the graph in semi-logarithmic coordinates, C3 and C4 do not pass the field emission Forbes test. The upper limit of the dimensionless field *f* in the test for compliance with the field operation mode for the work function of 4.6 eV exceeds *f* = 0.45 (insert in Figure 6i,l). This means that for emissive structures, the specified electric fields are extremely high and the cathodes operate almost at the limit of their capabilities.

**Figure 7:**
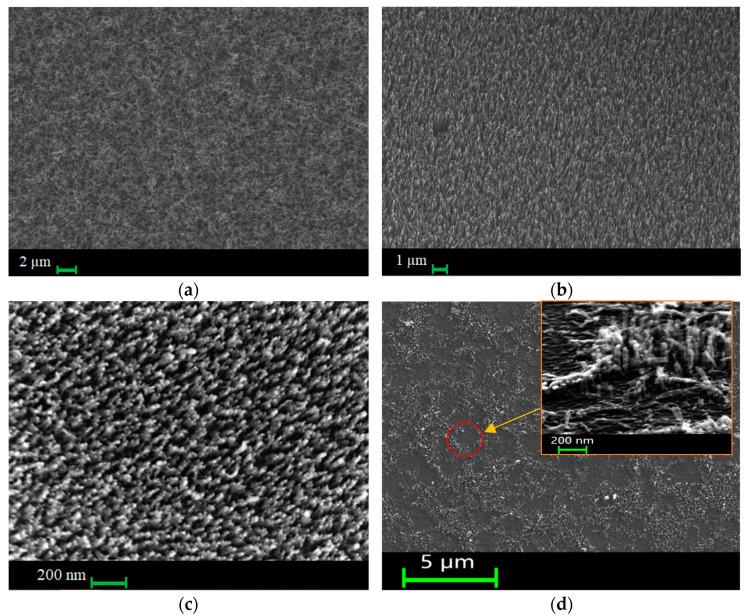
SEM images of samples C2 (**a**), C3 (**b**), and C4 (**c**). SEM images of sample C1 after the emission experiment: (**d**) general view of the emitter with the formed bald spots in the CNT array and a detailed view of the damage at an angle of 30° (in insert).

After the emission tests, microscopy was repeated to assess the resistance of CNTs to strong electric fields. For samples with C2–C4, no noticeable morphological changes in the nanotube arrays were detected after testing the cathodes (Figure 7a–c). However, there is a significant drawback to the array of short discrete nanotubes from C1. Since CNTs tend to polarize in an external electric field, the force acting on the CNTs from strong fields during emission can tear them off the substrate if the strength of their bond with it is less than the force acting from the external electric field. Figure 7d shows an SEM image of the structure with short CNTs after the emission tests, from which it can be seen that there are bald spots in the array of tubes, which were not there before the measurements of emission currents. A more detailed image in the insert for Figure 7 shows that CNTs lie on the substrate or are completely absent. The problem of the weak bonding of nanotubes with the substrate is still relevant. Perhaps the stability of dense arrays of CNTs is associated with the manifestation of the mutual screening effect achieved in dense arrays, as a result of which the magnitude of the electric field focused on the CNTs is significantly lower. The CNTs in the C4 structure also turned out to be resistant to exfoliation, since the array was denser compared to C1. Moreover, the overall tube array located under the highly protruding CNTs can also protect them from strong fields by partially shielding them from below. In the case of C1, the field acting on the CNTs is much stronger, since the mutual shielding of the tubes is minimal.

## 4. Conclusions

The comparison of various CNTs morphologies makes it possible to determine the direction of further work on optimizing CNT arrays to achieve the best performance in terms of the drawn current and uniform distribution of emission centers. This paper presents a production technology for different arrays of CNTs using DC PECVD on a Si substrate, a detailed analysis of their structure, and a comparison of their field emission properties. The studies showed a high degree of chemical purity of the synthesized CNTs under the presented technological conditions. In this work, we compared CNT arrays of different morphologies using a set of criteria, such as threshold field emission voltages, emission currents, the number and uniformity of the distribution of emission centers over the cathode area, as well as compliance with the normal current regime according to the “orthodox” field emission test.

The thickest CNT coating (C2) had the smallest turn-on field *E_on_* = 3.0 V/µm and threshold field *E_th_* = 2.3 V/µm, and had the highest field enhancement factor *β_eff_* = 2821, capable of producing 2.88 mA/cm^2^ at 4.53 V/µm. A rather good result showed that sample C4 had a current density of 2862.9 µA/cm^2^ at 11.5 V/µm. The worst result was shown by the structure with a continuous array of C3 vertical tubes, in which turn-on field (*E_on_* = 9.8 V/µm) and threshold field (*E_th_* = 9.0 V/µm) turned out to be the largest among the presented arrays, and the maximum recorded current density was only 93.5 µA/cm^2^, with a relatively low *β_eff_* = 617. It is possible that the effect of mutual screening in arrays of this type is responsible for the increase in switching fields, the decrease in the field gain, and, accordingly, the low emission currents.

The samples with short CNTs (C1 and C4) performed better in terms of the distribution uniformity of the emission centers and the uniformity of the current distribution. The uniformity of the distribution of emission centers for C2 was somewhat worse, and for C3, the assessment of the uniformity of the distribution of centers over the area was not at all applicable.

We checked whether the current mode of the samples corresponds to the classical law of field emission using the Forbes test (the so-called “orthodox” test). Samples C1 and C2 successfully passed the test, so the electron emission in the experiments corresponds to the FN law as the dimensionless field *f* = 0.4 does not exceed the value *f* set for CNTs (for the work function 4.6 eV) equal to 0.45. This means that the specified electric fields for these structures lie within acceptable ranges and correspond to the normal current regime. However, as can be seen from the processing of the graph in semi-logarithmic coordinates, C3 and C4 do not pass the field emission Forbes test. The upper limit of the dimensionless field f in the test for compliance with the field operation mode for the work function of 4.6 eV exceeds *f* = 0.45. This means that for emissive structures, the specified electric fields are extremely high and the cathodes operate almost at the limit of their capabilities.

The CNTs of the C1 sample are prone to tearing out in strong electric fields, which did not happen in the C2–C4 samples. To put into operation discrete arrays of tubes, it is necessary to overcome the obstacle associated with the detachment of tubes from the substrate. To achieve this, it is necessary to search for layers that strengthen their bond at the point of contact. Considering the resistance of a continuous array with a thick CNT coating to the action of an electric field, fairly good uniformity of the distribution of emission centers, a fairly high output current, and a normal current regime, the morphology of such structures can be suitable for various practical applications.

## Figures and Tables

**Table 1:** Field emission parameters of CNT arrays.

No.	Sample Description	*E_th_*, V/µm	*E_on_*, V/µm	*E_max_*, V/µm	*J_max_*, µA/cm^2^	*β_eff_*
C1	Discrete CNTs (length 200 nm)	6.5	8.1	11.6	936.6	1100
C2	Thick-cover CNTs (length 20 µm)	2.3	3.0	4.53	2880	2821
C3	Dense CNTs (length 7 µm)	9.0	9.8	13.3	93.5	617
C4	Dense CNTs (length 300 nm)	4.8	6.1	11.5	2862.9	1380

## Data Availability

The data presented in this study are available on request from the first author.
